# Psoas Abscess Presented as Right Hip Pain in a Young Adult With Crohn’s Disease

**DOI:** 10.7759/cureus.13162

**Published:** 2021-02-05

**Authors:** Sotirios G Doukas, Keshav Bhandari, Kim Dixon

**Affiliations:** 1 Department of Forensic Sciences and Laboratory of Toxicology, University of Crete Medical School, Giofirakia, GRC; 2 Department of Medicine, Saint Peter's University Hospital, New Brunswick, USA; 3 Department of Medicine, Saint Peter’s University Hospital, New Brunswick, USA

**Keywords:** psoas abscess, crohn’s disease (cd), hip pain

## Abstract

A 27-year-old man with a past medical history of Crohn's disease presented in the Emergency Department complaining of right hip pain that has been going on for one month. At presentation, the patient was tachycardic. Physical examination revealed a positive psoas sign. Laboratory tests showed elevated white blood cells, C-reactive protein, and erythrocyte sedimentation rate. Computed tomography of the abdomen and pelvis revealed ileo-psoas fistula and psoas abscess. This rare case aims to provide awareness that intra-abdominal pathology should always be suspected in patients with referred hip pain and Crohn's disease. A thorough physical examination including maneuvers for assessment of possible iliopsoas inflammation should be effectively performed at the bedside to determine the likelihood of the condition and proper imaging should follow to confirm the diagnosis.

## Introduction

Crohn's diseases often have intraabdominal complications like abscess formations. Psoas abscess formation in patients with Crohn's disease can be insidious. Here we present a case of psoas abscess in a patient with Crohn's disease, which presented as referred hip pain and we discuss the importance of abdominal examination in a patient with Crohn disease for the assessment of a possible psoas abscess.

## Case presentation

**Figure 1:**
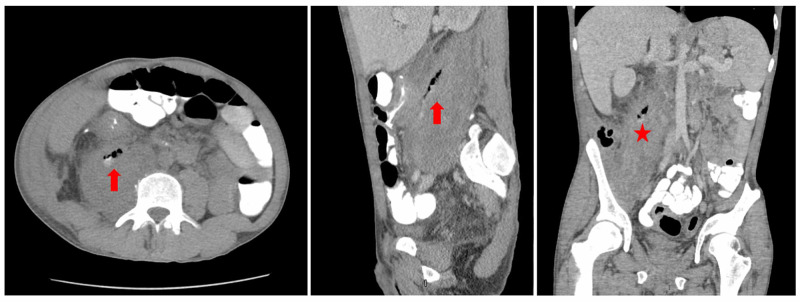
Computed tomography of the abdomen with and without oral and intravenous contrast showing a fistulous connection between the terminal ileum and the right psoas major (star). This was markedly edematous and inflamed and contained phlegmon/abscess (arrow).

A 27-year-old man with a past medical history of Crohn's disease presented in the Emergency Department with a complaint of a right hip pain for one month that has been worsening. The patient said that he visited his primary care physician weeks prior and was referred for physical therapy. The patient was compliant with physical therapy and received cyclobenzaprine; however, his pain did not improve. During examination, the patient pointed that the pain was localized to the right flank and right hip crest, sharp in nature, and intermittently radiating to the right lower quadrant. The patient reported that although he was able to bear weight, the pain was exacerbated with movement. The patient reported no fevers or chills, as well as no redness, or swelling, over the hip joint. The patient also had been having three to four soft bowel movements per day and reported that this is his baseline. The patient did not have any black stools, bright red blood in stools, nausea, or vomiting. Review of systems was also negative for dysuria, hematuria, and penile discharge (Figure 1).

The patient was diagnosed with Crohn's disease at age 11 years old and has been on ustekinumab 90mg every eight weeks for the last three years. The patient reported that he had a flare four months prior because he ran out of ustekinumab for three months due to the inability to cover the cost. The patient also had known small bowel resection and simultaneous appendectomy six years ago. The last colonoscopy the patient had was four years prior to the presentation and did not show any acute findings.

At presentation, the patient had a sublingual temperature of 99.8 F, with a blood pressure of 141/78 mmHg, a pulse of 130 beats per minute, a respiratory rate of 18 breaths per minute, and oxygen saturation of 100% on room air. On physical examination, the patient was not in acute distress. The abdominal examination revealed tenderness over the right lower quadrant, involuntary guarding, and hyperactive bowel sounds. Examination of the hip joint showed no redness, edema, or tenderness. No limitation in anterior hip flexion, lateral hip flexion, or internal rotation. Also, the costovertebral angle was not tender on percussion. Mild tenderness noticed on the external rotation of the right hip and the psoas sign was positive. Cardiovascular and pulmonary examinations did not produce significant findings. Laboratory investigations showed white blood cells of 15 x 10^3^/cu mm, hemoglobin 7.6 g/dL, MCV 73.5%, MCHC 32.0%, platelets 457 x 10^3^/cubic mm, reticulocyte count 1.3%, ESR 118 mg/L, C-reactive protein (CRP) 58 mg/L.

Given the suspicion for psoas abscess, blood cultures were sent and the patient was started on ampicillin and sulbactam. CT of the abdomen with and without oral and intravenous contrast was performed. This showed marked inflammation of the terminal ileum with the fistulous connection between the terminal ileum and the right psoas major, which was markedly edematous and inflamed, containing an abscess. A smaller abscess in the right iliacus muscle was also described. After the CT findings were reviewed, general surgery was consulted and antibiotics changed to metronidazole, cefepime, and vancomycin. Finally, interventional radiology performed abscess drainage.

## Discussion

Psoas abscess is defined as the collection of pus in the compartment of the iliopsoas muscle and is a rare and often misdiagnosed condition. The incidence is usually low, 0.4 per 100,000 people [1], however recent studies support that a significant number of cases are underreported and the diagnosis is usually challenging given the non-specific presentation [2]. The formation of psoas abscess can be due to lymphatic or hematogenous spread of infection, usually in patients with chronic conditions such as Diabetes Mellitus, chronic kidney disease, drug abuse, or immunosuppression. However direct spread and formation of the abscess are possible in cases like Crohn's disease, appendicitis, pancreatitis, diverticulitis, urinary tract infections, osteomyelitis of vertebral bodies, or spondylodiscitis [3].

The transmural inflammation of Crohn's disease increases the risk for bowel perforation and formation of fistula that can lead to psoas abscess formation. Although psoas abscess is most commonly present in longstanding Crohn's disease, it can also be its first manifestation [4,5].

The symptomatology of psoas abscess can vary and as a result, the diagnosis might be delayed until signs of sepsis have established. Fever, flank pain, and hip pain often with antalgic gait are the classic symptoms, however, only 30% of the patients with poas abscess initially present with this triad [2,4,6,7]. As we see in this case, referred hip pain in patients with long-standing Cohn disease should always raise the suspicion for ongoing psoas abscess formation [8-10].

The underlying pathophysiology of referred pain to the anterior thigh in iliopsoas abscess involves the irritation of the femoral nerve and specifically of an intermediate femoral cutaneous nerve that provides sensory innervation of the anterior aspect of the thigh [4]. This radiation of the pain to the anterior thigh instead of the lateral or posterior aspect of the leg should increase the suspicion that the underlying pathology is less likely to involve sciatica or joint [4].

Physical examination is crucial in cases of suspected intraabdominal pathology [11]. Psoas extension test is also known as Cope’s psoas test, is a recommended approach to diagnosing ongoing inflammatory pathology of the psoas muscle [4]. Psoas extension test is performed by having the patient lie supine on the non-affected side, and hyperextending the hip joint [4]. The test is considered positive when irritation or pain is induced by stretching and passively extending the iliopsoas muscle [4]. The patient in our case had a positive psoas sign further supporting the probability for ongoing iliopsoas inflammatory pathology.

Laboratory investigations in cases of psoas abscess often reveal signs of ongoing inflammation. An increase in white cell count, CRP, and erythrocyte sedimentation rate is commonly found [12]. However, the sensitivity and specificity of these tests are not significant [12].

Imaging is necessary to confirm the diagnosis of psoas abscess. Although bedside ultrasound is a relatively inexpensive and safe approach, the success rates are relatively low for the detection of psoas abscess [13]. The preferred test is CT abdomen and pelvis with contrast [8,13,14]. In this case, the CT abdomen and pelvis with and without oral and IV contrast reveal an ileuopsoas fistula and psoas abscess. The formation of external and internal fistulas is not an uncommon finding. In line with our case, Christodoulou et al. also reported a case of a young male with hip pain found to have Crohn’s disease with entero-psoas fistula and psoas abscess supporting that clinicians should be aware that hip pain in patients with Crohn's disease may indicate intraabdominal pathology [15].

Without any doubt, tremendous progress has been made in the development of accurate and reliable laboratory and imaging tests for the diagnosis of acute and chronic abdominal pathologies. However, the reported decline in physicians’ bedside skills has been a reality with multiple studies supporting that reinforcing clinical skill education as part of medical training is a necessity.

## Conclusions

This study presents a case of a Crohn's disease complaining of hip pain and diagnosed with psoas abscess, highlighting the importance of physical examination for the diagnosis of this insidious disorder. Overall, suspicion of intraabdominal pathology and specifically for psoas abscess formation should always be suspected in patients with Crohn's disease and hip pain or antalgic gait. Psoas sign has a high specificity and can be used to support the differential.
